# Blastic Plasmacytoid Dendritic Cell Neoplasm: A Case Report

**DOI:** 10.7759/cureus.37016

**Published:** 2023-04-01

**Authors:** Abdulrahman Nasiri, Arwa Lami, Alanoud Alhumaidi, Afnan Madkhali, Amnah Althaqib, Norah Aljarwan, Reem Alkharras

**Affiliations:** 1 Hematology, King Faisal Specialist Hospital & Research Centre, Riyadh, SAU; 2 Internal Medicine, Security Forces Hospital, Riydah, SAU; 3 Internal Medicine, Security Forces Hospital, Riyadh, SAU

**Keywords:** azacytidine, venetoclax, bcl-2, cd123, rare, bpdcn, neoplasm, dendritic, plasmacytoid, blastic

## Abstract

Blastic plasmacytoid dendritic cell neoplasm (BPDCN) is an uncommon hematological tumor originating from the precursor of plasmacytoid dendritic cells (pDCs) with a persistent and progressive course of illness. Despite being an aggressive disease BPDCN has an initial indolent course manifested as skin lesions. Alongside or following the skin lesion, the extra-cutaneous manifestation develops and includes lymphadenopathy, splenomegaly, and hepatomegaly. The BPDCN diagnosis is mainly based on the immunophenotype.

Herein, we report the case of a 72-year-old male patient who presented with a history of left anterior chest wall painless skin lesions. Histology of skin biopsy of the left chest skin lesion showed diffuse dermal infiltration by monomorphic medium-sized blastic cells positive for cluster of differentiation (CD)4, CD45, CD7, CD56, CD43, CD123, T-cell leukemia-1 (TCL1), and B-cell leukemia/lymphoma 2 protein (BCL2). Given the rarity of the disease, standard chemotherapy regimens used in treating different leukemias and lymphomas have been adapted to treat BPDCN.

## Introduction

Blastic plasmacytoid dendritic cell neoplasm (BPDCN), an uncommon hematological tumor originating from the precursor of plasmacytoid dendritic cells (pDCs), has a persistent and progressive course of illness [1]. It accounts for only about 0.44% of new hematologic malignancies annually. The exact etiology of the disease is not well understood. However, an association with other hematological malignancies has been observed.

While BPDCN is an aggressive disease, it initially has an indolent course manifested as skin lesions. Extra-cutaneous manifestations such as lymphadenopathy, splenomegaly, and hepatomegaly develop alongside or after the skin lesion, [2].

The diagnosis of BPDCN is primarily determined by the immunophenotype. As this is a rare disease, standard chemotherapy regimens for a variety of leukemias and lymphomas have been adapted to treat it. However, the perception of this systemic condition has grown and led to the development of various novel targeted pharmacological agents.

## Case presentation

Patient information

We report the case of a 72-year-old male patient with type 2 diabetes mellitus being treated with metformin and dyslipidemia on atorvastatin. The patient came to our attention after presenting to the dermatology clinic with a four-month history of left anterior chest wall painless skin lesions. The patient denied any history of fever, night sweats, weight loss, recurrent infection, symptoms of anemia, bleeding, mouth ulcer, joint pain or swelling, or respiratory or gastrointestinal symptoms.

Clinical findings

**Figure 1:**
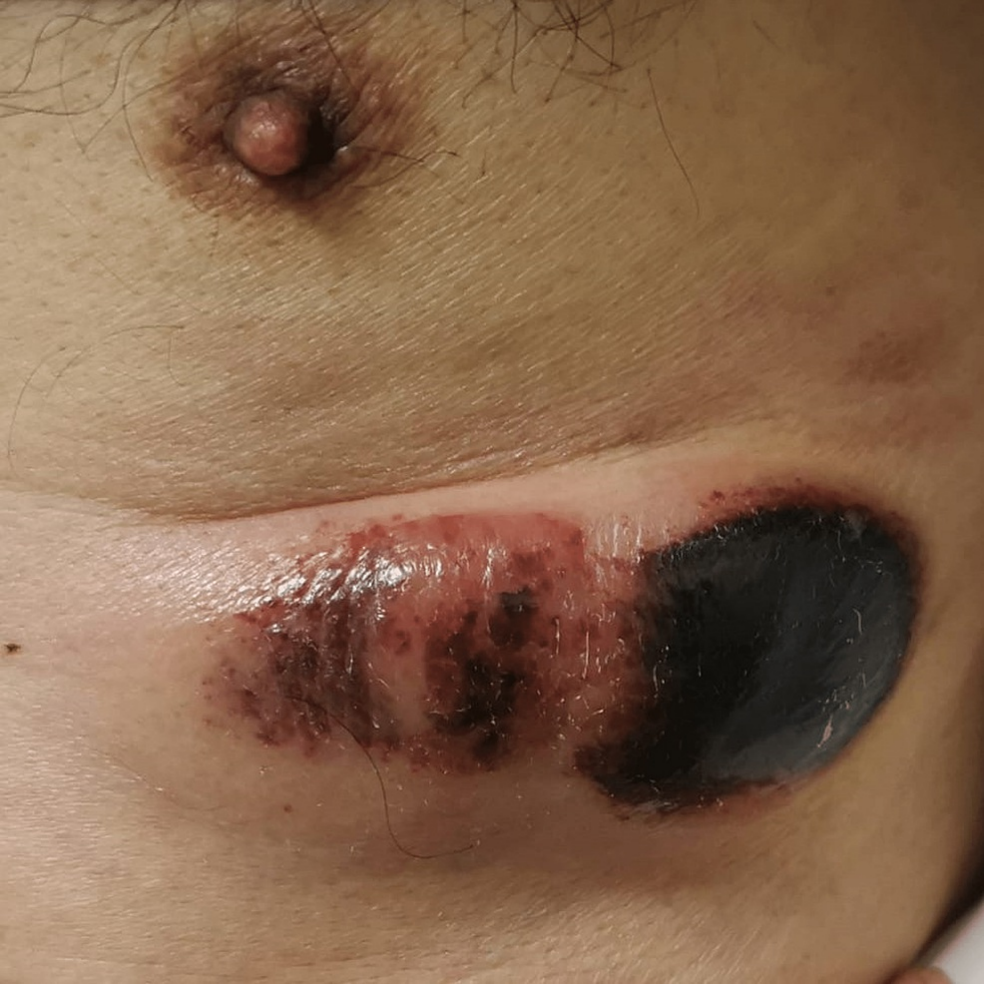
Left anterior chest wall oval lesion measuring around 10 cm x 5 cm with violaceous hemorrhagic in appearance, and painless.

Examination showed an oval left anterior chest wall lesion measuring around 10 cm x 5 cm and located immediately below the left nipple. It was violaceous hemorrhagic in appearance and painless (Figure 1). The remainder of the skin examination did not reveal other visible lesions and there was no palpable peripheral lymphadenopathy nor hepatosplenomegaly.

Diagnostic assessment

Complete blood count with differential showed a white blood count of 8.5X9/L, hemoglobin of 11.1 g/L, and platelets of 103 10^3 ^X μl. Corrected calcium was 2.23, the lactic acid dehydrogenase level was 185 U/L, the alkaline phosphatase level was 74 U/L, and the uric acid level was 411 µmol/L (Table 1).

**Table 1:** Laboratory tests HGB: Hemoglobin, HCT: Hematocrit, MCV: Mean cell volume, MCHC: Mean cell hemoglobin concentration, PLT: Platelets, ESR: Erythrocyte sedimentation rate, CRP: C-reactive protein, NA: Sodium, K: Potassium, CR: Creatinine, LDH: Lactic acid dehydrogenase, ALT: Alanine transaminase, AST: Aspartate transaminase, PT: Prothrombin Time, INR: International normalized ratio, ALP: Alkaline phosphatase, APTT: Activated partial thromboplastin time, TIBC: Transferrin iron-binding capacity

Lab	Result	Normal value
Complete blood count		
WBC (10^9^/L)	8.5	4.5-13.5
RBC (10^12^/L)	3.48	3.8-6.5
HGB g/L	11.1	11.5-180
HCT %	0.39	0.35-0.52
MCV fl	88.2	77-98
MCHC g/L	327.0	310-360
PLT 10^3 ^X μl	103	150-400
Inflammatory markers		
ESR mm/hr	12	0-20
CRP	47.66	Less 5.0
Electrolytes		
NA mmol/L	136	136-145
K mmol/L	3.4	3.5-5.1
UREA mmol/L	4.6	2.76-8.07
CR umol/l	40	62-106
Uric acid µmol/L	411	208 – 428
Lactic acid dehydrogenase		
LDH U/L	185	135-225
Liver function tests		
ALT U/L	13	UP TO 41
AST U/L	16	UP TO 40
ALP U/L	74	82-331
Bilirubin-total umol/l	4.6	0-17.1
Serum calcium mmol/L	2.23	2.25-2.62
Iron studies		
Iron umol/l	30.4	5.83-34.5
Transferrin g/L (TIBC)	1.51	2.0-3.6
Transferrin	81	15-45%
Coagulation profile		
PT (in seconds)	14.2	10.0-14.1
INR	1.23	0.86-1.2
APTT (in seconds)	38.9	24.6-40.1

Histology of the skin biopsy of the left chest skin lesion showed epidermis and underlying dermis with diffuse dermal infiltration by monomorphic medium-sized blastic cells with irregular nuclei, fine chromatin inconspicuous nucleoli, and scant cytoplasm. Scattered myositis was seen. No necrosis was observed. The immunostaining of the tumor cells was positive for cluster of differentiation (CD)4, CD45, CD7, CD56, CD43, CD 123, T-cell leukemia-1 (TCL1), and B-cell leukemia/lymphoma 2 protein (BCL2). And it was negative for CD20, CD 19, paired box protein 5 (PAX5), CD2, CD3, CD5, CD8, granzyme B (GrB), and CD45Ro (Table 2). These findings were diagnostic for blastic plasmacytoid dendritic cell neoplasm.

**Table 2:** Immunohistochemistry of the tumor cell CD: Cluster of differentiation, BCL:  B-cell leukemia/lymphoma 2 protein, Pax-5: Paired box protein 5, EBV: Epstein–Barr virus, ALK-1: Anaplastic lymphoma kinase

Positive	Negative
CD4, CD45, CD7, CD56, CD43. CD 123, TCL- I, and BCL2	CD20, CD 19, PAX5, CD2, CD3, CD5, CD8, Granzyme B (GrB), CD45Ro, EBV, CD10, BCL6, CD30, ALK-1, myeloperoxidase, CD99, CD68, CD 163, CD34, CD la, and CD117

Following this result, the patient underwent staging with CT and bone marrow aspiration, and biopsy. The CT scan of the chest abdomen, and pelvis demonstrated the infiltrative lesion in the skin of the left anterior chest wall with no intrathoracic or intraabdominal metastasis. The bone marrow aspiration and biopsy revealed hypercellular bone marrow (cellularity 50% for the patient's age). The megakaryopoiesis was adequate with mild dysmegakaryopoiesis. Active granulopoiesis and erythropoiesis were observed. There was no abnormal collection or increase of blasts.

Therapeutic intervention

The patient was started on venetoclax and azacytidine protocol. Azacytidine was given at 75 mg/m2 (days 1 to 7). Venetoclax was started with 100 mg per oral once daily and then escalated to 400 mg per oral once daily.

Follow-up and outcomes

**Figure 2:**
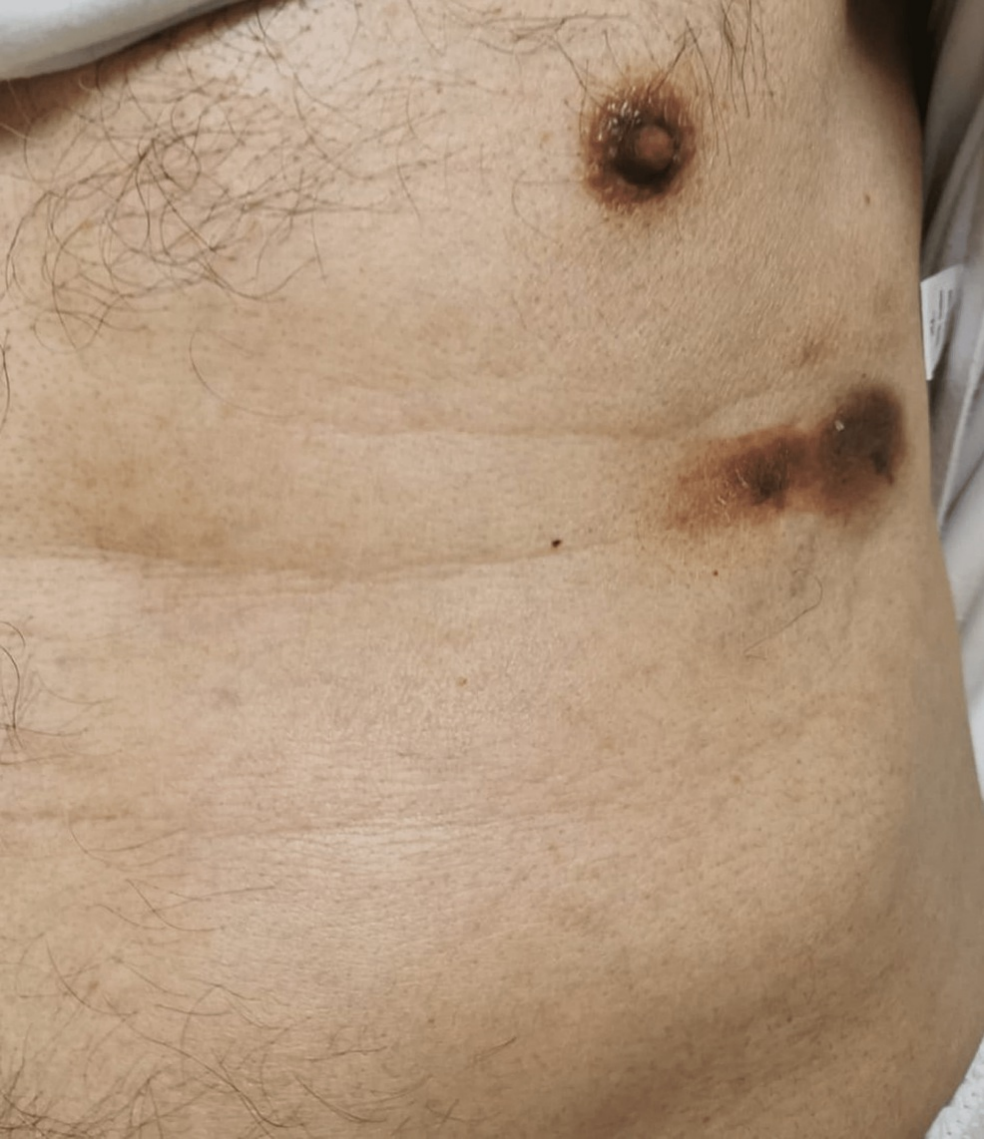
Remarkable reduction in the size of the lesion

**Figure 3:**
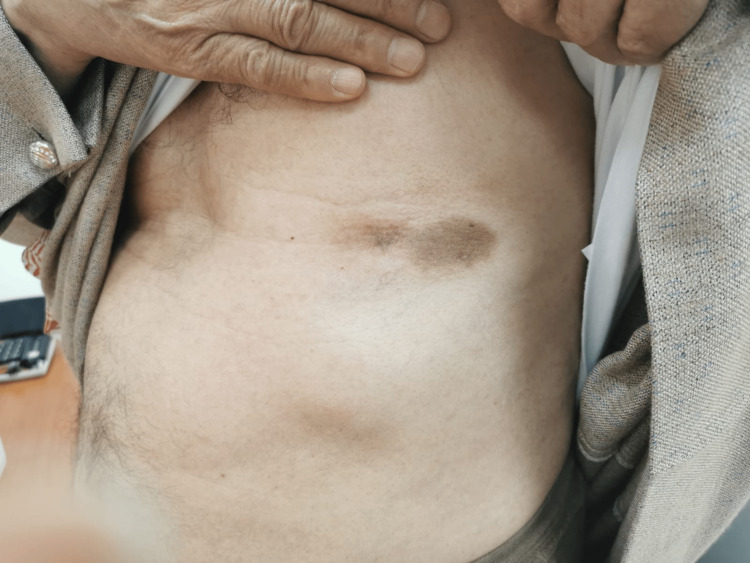
Further reduction in the size of the lesion

The patient tolerated the treatment regimen well without any evidence of tumor lysis syndrome or significant cytopenia. The skin lesion showed a remarkable reduction in size (Figures 2, 3).

## Discussion

The nomenclature used to describe BPDCN has evolved since it was first reported in 1994 as our understanding of the disease’s biology improved [2]. Initially, it was known as agranular CD4+ natural killer (NK) cell leukemia, then the term ‘blastic NK cell lymphoma’ was used. Later, the term ‘agranular CD4+CD56+ hematodermic neoplasm/tumor’ was deployed based on the immunophenotype and the tendency for skin involvement [3]. Currently, it is recognized as a different disease and separately cataloged in the group of acute myeloid leukemia (AML), and related to precursor neoplasms of the hematopoietic and lymphoid tissues as per the 2008 World Health Organization (WHO) classification of tumors [4].

Blastic plasmacytoid dendritic cell neoplasm accounts for only about 0.44% of new hematologic malignancies annually. The disease mainly affects elderly patients in their seventh or eighth decade of life with a clear male predominance (male/female ratio of 3:1), with some pediatric cases reported in the literature [5].

The exact etiology of the disease is not well understood. However, an association with other hematological malignancies was observed. The associated malignancies include myelodysplastic syndromes (MDS), MDS/myeloproliferative neoplasms (MPN), and chronic myelomonocytic leukemia (CMML) [6,7].

Though an aggressive disease, BPDCN begins with an indolent course that manifests as skin lesions. The skin lesions are asymptomatic, solitary, or multiple and might exhibit different patterns from nodular lesions to plaques or bruise-like areas, and have a size ranging from a few millimeters to several centimeters with no preferred anatomic area [1,5,8].

Our patient's demographic characteristics align with the typical presentation of BPDCN as he is a 72-year-old male with a skin lesion matching the reported skin lesion characteristics: violaceous hemorrhagic in appearance, and painless lesion. It is worthwhile to mention that the literature described a few cases lacking skin involvement with fulminant leukemia as the sole presentation [9].

Alongside or following the skin lesion, the extra-cutaneous manifestation develops. This includes lymphadenopathy, splenomegaly, and hepatomegaly [10]. Peripheral blood and bone marrow involvement vary greatly and results in cytopenia, with thrombocytopenia being the most common, followed by anemia and neutropenia [8,11].

Microscopically, BPDCN is described by a widespread, poorly differentiated monomorphous infiltrate of medium-sized blasts. Nuclei have a marginally irregular profile, fine chromatin, and from one to three small-scale nucleoli. Bone marrow biopsy shows either slight interstitial infiltrates or more commonly diffuse replacement [1,12].

Neoplastic cells constantly express high levels of CD123, CD4, human leukocyte antigen (HLA)-DR, and cutaneous T-cell lymphoma 1 (cTCL1). Blastic plasmacytoid dendritic cell neoplasm very often expresses CD56, CD36, CD38, CD303, CD304, and CD45RA. It is regularly negative for LAT, lysozyme, myeloperoxidase (MPO), CD3, CD13, CD16, CD19, and CD20. It may also express other antigens negative in normal pDCs, including interferon regulatory factor 4 (IRF4), BCL6, and BCL2 (the latter provides a therapeutic target in the affected individuals) [3,13,14]. In this case, the patient's tumor cells were positive for CD123, CD4, HLA-DR, and cTCL1, which is consistent with the diagnosis of BPDCN based on the typical immunophenotype.

Patients with BPDCN are affected by frequent chromosomal alterations. Hypodiploid or complex aberrant karyotypes with six to eight aberrations are frequently reported, but no changes specific to the disease are observed. Molecular genetic mutations are most frequently detected in the genes tet methylcytosine dioxygenase 2 (TET2), additional sex combs-Like 1 (ASXL1), tumor protein 53 (TP53), FMS‐like tyrosine kinase 3 (FLT3), Ikaros zinc finger 1 (IKZF1), neuroblastoma ras viral oncogene homolog (NRAS), and nucleophosmin 1 (NPM1) in genes of the Ikaros family and zinc finger E-box binding homeobox 2 (ZEB2) [8,12,15].

Inherent resistance to conventional chemotherapies is a feature of BPDCN. Most treatment responses are temporary, and the overall results are generally quite poor. Considering the rarity of the disease, standard chemotherapy regimens used in treating numerous leukemias and lymphomas have been adapted to treat BPDCN. However, the perception of this systemic condition has grown and led to the development of various novel targeted pharmacological agents including targeted agents for CD123, BCL2, and beyond [16,17].

Before the targeted-therapy era, treatment typically included regimens such as hyper-fractionated cyclophosphamide, vincristine, doxorubicin, and dexamethasone alternating with high-dose methotrexate and cytarabine (hyperCVAD), high-dose methotrexate with asparaginase (aspa-MTX), or cyclophosphamide, doxorubicin, vincristine, prednisone (CHOP) [8,17].

Many of these protocols have been successful in leading to complete remission (CR), with acute lymphoblastic leukemia (ALL) regimens chosen over acute myeloid leukemia (AML) regimens as they seem to be more effective in terms of response rates if patients can tolerate it, often followed by allogeneic or autologous hematopoietic stem cell transplantation (HSCT) [1,10]. Numerous data indicate improved outcomes in terms of long-lasting remissions and relapse rates with allogeneic stem cell transplantation (allo-SCT) compared to auto-SCT [18].

Blastic plasmacytoid dendritic cell neoplasm typically affects elderly patients, with a median age of 68 years, thus it should be emphasized that HSCT might not be feasible. Limited investigations address the treatment of elderly patients who are frail or transplant-ineligible. In such a population, the best supportive care should be emphasized, and palliative or hospice care procured early [17].

Reduced intensity regimens options include low-dose dexamethasone, VP16, ifosfamide, carboplatin (DeVIC); cyclophosphamide, vincristine, and prednisone (COP), which were used most frequently in patients presenting with the extracutaneous disease, followed by radiation therapy in limited cutaneous disease as well as a few additional treatments such as hydroxyurea, 6-mercaptopurine-methotrexate-prednisone, etoposide-prednisone, or prednisone alone [19].

Single agents demonstrated some efficacy in BPDCN including pralatrexate, bendamustine, or gemcitabine/docetaxel combinations. Although the results were encouraging, these trials were only conducted on a small number of individuals, and they need to be confirmed in larger trials [1,17,20]. Furthermore, a study of two patients has shown the utility of the hypomethylating agent azacitidine in BPDCN with an excellent response after a single cycle [21].

In the current era of targeted therapy, therapeutic agent targeting CD123 have shown favorable activity including tagraxofusp-erzs (SL-401) (approved in December 2018), and IMGN 632 (FDA breakthrough designation, October 2020, for relapsed/refractory BPDCN) [22]. Chimeric antigen receptor T-cell therapy (CAR-T) has displayed effectiveness in several hematologic malignancies and despite being in the early days of investigation in the field of BPDCN, it led to the development of CD28/4-1BB anti-CD123 which demonstrated a sustained complete response (CR) in a single patient with recurrent BPDCN after allogeneic HSCT. More studies utilizing anti-CD123 CAR-T cell therapy are underway for relapsed/refractory BPDCN (NCT02159495 and NCT03203369) [23].

Another potential therapeutic target is BCL2 frequently overexpressed in BPDCN. Venetoclax, an inhibitor of BCL2 demonstrated response and durable CR in several reports in patients with relapsed BPDCN [23]. Due to the frailty of the reported patient, a tolerable treatment regimen with an acceptable safety profile was necessary to manage the disease. The positive expression of BCL2 in the patient allowed for such a regimen to be implemented and effectively control the disease.

Several studies have looked into BPDCN treatment plans implemented for multiple myeloma. Lenalidomide has been effective in preclinical trials involving BPDCN cell lines in mice. Additionally, a case report has demonstrated that daratumumab, a monoclonal antibody that targets CD38, has shown promise in producing a positive clinical response [22].

Regardless of initial CR, overall survival (OS) is inadequate, and patients typically relapse within two years most often in the skin, bone marrow, or central nervous system (CNS). If BPDCN relapse occurs, the disease has been observed to be aggressive and resistant. However, there have been instances of significant success in achieving partial remission by administering re-induction chemotherapy followed by a repeat HSCT. The advancement of novel targeted therapeutic agents has hastened the disease course in relapse [1,8,17,22].

## Conclusions

Blastic plasmacytoid dendritic cell neoplasm is a rare and aggressive hematological tumor originating from precursor cells. It mainly affects elderly males and presents with skin lesions that progress to extra-cutaneous manifestations. Diagnosis is based on the immunophenotype, with neoplastic cells expressing CD123, CD4, HLA-DR, and cTCL1. Blastic plasmacytoid dendritic cell neoplasm has inherent resistance to conventional chemotherapies but targeted pharmacological agents such as CD123 and BCL2 inhibitors, show promise.
